# Importin-β From the Recretohalophyte *Limonium bicolor* Enhances Salt Tolerance in *Arabidopsis thaliana* by Reducing Root Hair Development and Abscisic Acid Sensitivity

**DOI:** 10.3389/fpls.2020.582459

**Published:** 2021-01-13

**Authors:** Yanyu Xu, Xiangmei Jiao, Xi Wang, Haonan Zhang, Baoshan Wang, Fang Yuan

**Affiliations:** Shandong Provincial Key Laboratory of Plant Stress, College of Life Sciences, Shandong Normal University, Jinan, China

**Keywords:** ABA sensitivity, *Arabidopsis thaliana*, *Limonium bicolor*, root hair, salt stress, trichome

## Abstract

**Aims:**

To elucidate the genetics underlying salt tolerance in recretohalophytes and assess its relevance to non-halophytes, we cloned the *Limonium bicolor* homolog of *Arabidopsis thaliana* (Arabidopsis) *SUPER SENSITIVE TO ABA AND DROUGHT2* (*AtSAD2*) and named it *LbSAD2*, an importin-β gene associated with trichome initiation and reduced abscisic acid (ABA) sensitivity, and then we assessed the heterologously expressed *LbSAD2* in Arabidopsis.

**Methods:**

We examined *LbSAD2* expression and assessed the effect of heterologous *LbSAD2* expression in Arabidopsis on root hair/trichome induction; the expression levels of possible related genes in trichome/root hair development; some physiological parameters involved in salt tolerance including germination rate, root length, and contents of Na^+^, proline, and malondialdehyde; and the response of ABA at the germination stage.

**Results:**

The *LbSAD2* gene is highly expressed in the salt gland development stage and salt treatment, especially located in the salt gland by *in situ* hybridization, and the LbSAD2 protein contains some special domains compared with AtSAD2, which may suggest the involvement of LbSAD2 in salt tolerance. Compared with the *SAD2/GL1* mutant CS65878, which lacks trichomes, *CS65878-35S:LbSAD2* had higher trichome abundance but lower root hair abundance. Under 100 mM NaCl treatment, *CS65878-35S:LbSAD2* showed enhanced germination and root lengths; improved physiological parameters, including high proline and low contents of Na^+^ and malondialdehyde; higher expression of the salt-tolerance genes Δ*1-PYRROLINE-5-CARBOXYLATE SYNTHETASE 1* (*P5CS1*) and *GST CLASS TAU 5* (*GSTU5*); reduced ABA sensitivity; and increased expression of the ABA signaling genes *RESPONSIVE TO ABA 18* (*RAB18*) and *SNF1-RELATED PROTEIN KINASE 2* (*SRK2E*), but not of the ABA biosynthesis gene *9-CIS-EPOXYCAROTENOID DIOXYGENASE 3* (*NCED3*).

**Conclusion:**

*LbSAD2* enhances salt tolerance in Arabidopsis by specifically reducing root hair development, Na^+^ accumulation, and ABA sensitivity.

## Introduction

The global demand for land resources is increasing with the continuous expansion of the world population (Munns and Tester, 2008; Ma et al., 2020), and the increasing demand for food is driving the need for high crop yields. Soil salinization reduces crop yields and is an increasingly serious problem among the factors restricting food production (Boyer, 1982; Yuan et al., 2013). Rising salinity already affects 800 million hectares of land, and irrational irrigation methods have led to a rapid expansion of saline–alkali soils around the world (Munns and Tester, 2008; Song et al., 2020; Zheng et al., 2020). Few crops can survive in salt-affected areas, which has led to a significant decline in yield and caused soil degradation and desertification (Flowers and Colmer, 2008; Song and Wang, 2015). Developing methods to grow crops on saline lands has emerged as an important research topic in agriculture (Song et al., 2017; Han et al., 2020a). One promising method to improve crop yield on high-salt soils is transforming salt-sensitive plants with genes that confer salt tolerance (Yuan et al., 2018); halophytes are promising sources for such genes (Guo et al., 2020b).

Halophytes can complete their life cycles at ≥200 mM NaCl because they can secrete salt to the outside environment (recretohalophytes), sequester salt ions into the cell vacuole (euhalophytes) (Guo et al., 2020a), or block salt from entering the cells (pseudohalophytes) (Yuan et al., 2019a). The genomes of halophyte plants represent important genetic resources for efforts to improve salt tolerance in crop plants. *Limonium bicolor* is a typical recretohalophyte and excretes excess salt ions through its unique salt glands (Ding et al., 2010; Deng et al., 2015). The salt gland is a specialized plant epidermal structure, which can be easily visualized based on its blue autofluorescence under a fluorescence microscope. Yuan et al. (2014) explored the possible differentiation mechanisms of the *L. bicolor* salt glands and categorized five stages of their development during the differentiation of true leaves, as well as their ultrastructure (Feng et al., 2014, 2015). Numerous genes and transcription factors were identified that were differentially expressed during the course of salt gland development, including many with high similarity to genes involved in the trichome development pathways of other plants (Yuan et al., 2015).

Epidermal hairs serve as a physical barrier against biological stress, and their development has been well studied in Arabidopsis. In particular, the salt gland development transcriptome data indicated that the control of *L. bicolor* salt gland differentiation involved many homologs of the genes involved in initiating the development of Arabidopsis epidermal hairs (trichomes), such as *GLABRA1* (*GLABROUS1*, *GL1*), *TRANSPARENT TESTA GLABRA1* (*TTG1*), *GLABRA3* (*GL3*), *ENHANCER OF GLABRA 3* (*EGL3*), *SUPER SENSITIVE TO ABA AND DROUGHT2* (*SAD2*), *TRIPTYCHON* (*TRY*), and *CAPRICE* (*CPC*). Given that *L. bicolor* does not develop trichomes (Leng et al., 2018) and given the similar excretory/secretory functions of salt glands and trichomes and the same developmental stages of the first differentiated epidermal structure (Yuan et al., 2015), we speculate that *L. bicolor* salt glands may follow a developmental pathway similar to that of trichomes.

To test this, we characterized a *L. bicolor* importin-β protein (Lb125774, LbSAD2), which showed high similarity with AtSAD2, which functions as a positive upstream regulator during the development of Arabidopsis trichomes (Wang et al., 2011). *AtSAD2* deletion increases plant sensitivity to the phytohormone abscisic acid (ABA), and AtSAD2 is a β-domain protein that may participate in nuclear transport, thus influencing ABA sensitivity (Verslues et al., 2006). The *CS65878* mutant shows less DNA damage from UV-B treatment than the wild type (Zhao et al., 2007). During Arabidopsis trichome development, *AtSAD2* mediates the function of *AtGL3* in regulating the expression of *AtGL1*, *AtTTG1*, and *AtGL2* (Gao et al., 2008; Wang et al., 2011).

Here, we investigated the function of *LbSAD2*, which was highly expressed during salt gland development. We also tested its function in salt tolerance by heterologously expressing it in Arabidopsis, which rescued the ABA insensitivity and trichome defects of Arabidopsis *CS65878* mutants, but was also associated with reductions in root hair number (unlike *AtSAD2*). Heterologous expression of *LbSAD2* in wild-type (named as WT throughout the text) Arabidopsis increased plant salt tolerance, affecting root hair development, ABA sensitivity, and salt-related gene expression; taken together, these results suggest that *LbSAD2* may be useful for improving plant salt tolerance.

## Materials and Methods

### Plant Materials and Growth Conditions

Inflorescence *L. bicolor* were collected from a saline inland environment (N37°20′; E118°36′) in the Yellow River Delta, Shandong, China. Dried seeds were stored in a refrigerator at 4°C until needed. Before planting, seeds were surface-sterilized in 70% ethanol for 5 min, and then the ethanol was poured out, 6% (v/v) sodium hypochlorite (Sigma, United States) was added, and the seeds were vigorously shaken for 15–20 min. The seeds were washed thoroughly with sterile distilled water and then germinated on Murashige and Skoog (1962) medium (MS medium; adjusted to pH 5.8 with KOH before being autoclaved). Seeds were cultured at 28 ± 3°C/23 ± 3°C (day/night) at a light intensity of 600 μmol/m^2^/s (15-h photoperiod) and 70% relative humidity. The first true leaves were collected in liquid nitrogen at the undifferentiation stage (stage A, ∼5,000 leaves) and salt gland development stage (stage B, ∼4,000 leaves) (Yuan et al., 2015), and their RNA was extracted for gene cloning.

The Arabidopsis ecotype Columbia-0 (Col-0) was used as a control. The mutant CS65878 in double deletion of both AtGL1 (AT3G27920) and AtSAD2 (AT2G31660) (hereafter referred to as *CS65878*) was ordered on Arabidopsis Biological Resource Center. The seeds of Col-0 and *CS65878* Arabidopsis were sterilized with 75% ethanol for 4 min, repeated three times, during which time they were thoroughly vortexed, then sterilized with 95% ethanol for 1 min, repeated three times, and finally washed with sterile water four times. The seeds were planted on 1/2 MS medium (pH 5.8). After 2 days of vernalization at 4°C, seeds were cultured at 22°C/18°C (day/night) under a 16-h/8-h light/dark cycle with a light level of 150 μmol/m^2^/s and 70% relative humidity (Sui et al., 2017). After 1 week of culture, seedlings were transplanted to pots (10 cm in diameter and 8 cm in height) containing well-mixed soil (soil:vermiculite:perlite, 3:1:1) for further flowering and transformation.

### Full-Length Cloning and Bioinformatics Analysis of *LbSAD2*

The first true leaves of *L. bicolor* plants were collected at stages A and B and stored at –80°C (Yuan et al., 2015) and their total RNA was extracted. Template cDNA was obtained by reverse transcription using ReverTra Ace qPCR RT kit (TOYOBO, Japan). With reference to the Iso-seq transcriptome (unpublished) of *L. bicolor*, primers (*Lb*SAD2-S and *LbSAD2*-A) of Lb125774 (*LbSAD2*) were designed to clone the full-length coding sequence using Primer Premier 5.0 (Supplementary Table 1).

Alignment was performed using DNAman and DNAstar to compare nucleic acid and protein sequences. After NCBI BLASTp using LbSAD2, 33 SAD2 proteins were chosen for a phylogenetic tree construction using the neighbor-joining method with MEGA5.1^1^ and ClustalX, with statistical support for nodes obtained from at least 1,000 trials. The ProtParam tool in the ExPASy online software was used to predict the physical and chemical properties of the protein based on its primary structure^2^. The Prot-Scala tool in ExPASy was used to analyze the hydrophilicity and hydrophobicity of the amino acid sequence^3^. The signal peptide was detected with Signal4.1^4^, the GOR4 tool in ExPASy was used to predict the secondary structure of the protein^5^, SMART software was used to predict the conserved domain of the protein^6^, and SWISS-MODEL in ExPASy was used to predict the tertiary structure^7^.

### Subcellular Localization of *LbSAD2* by Transient Expression in Onion Epidermal Cells

The open reading frame (ORF) region of *LbSAD2* was cloned into the vector pCAMBIA1300, containing a CaMV 35S promoter, hygromycin resistance gene, and GFP reporter gene, by homologous recombination with the primers *LbSAD2* 1300-S and *LbSAD2* 1300-A (Supplementary Table 1) using the In-Fusion HD Cloning Kit (Clontech Laboratories, Inc.). The resulting p1300-*LbSAD2* vector was transformed into onion epidermal cells using *Agrobacterium tumefaciens* strain GV3101 (Sun et al., 2007). Fluorescence signals of labeled LbSAD2 were detected by microscopy (TCS S8 MP two-photon laser scanning confocal microscope, Leica, Germany). Simultaneously, the nucleus was positioned using DAPI staining observed under 358 nm excitation. The plasma membrane was stained using N-(3-triethylammoniumpropyl)-4-(6-(4-(diethylamino)phenyl)hexatrienyl (FM4-64, pyridinium dibromide, Invitrogen) and excited under 559 nm.

### Expression of *LbSAD2* in *L. bicolor* at Different Developmental Stages and Conditions

Previous RNA sequencing (RNA-seq) data for *L. bicolor* samples at different developmental stages (Yuan et al., 2015) indicate that *LbSAD2* expression varies among different stages. To further verify the differences in gene expression in different developmental stages and conditions, a variety of sample materials were obtained, including the first true leaves at stages A and B (undifferentiated stage, 4–5 days after sowing and salt gland differentiation stage, 6–7 days after sowing, using ∼4,000 leaves), stage C and D leaves (stomata differentiation stage, 8–10 days and pavement cell differentiation stage, 11–13 days, ∼2,000 leaves), stage E leaves (mature young stage, >14 days, ∼500 leaves), and old leaves (>20 days); stage E petioles and roots; and leaves collected after treatment for 14 days with 0.1 mg/L ABA, 24 h with 1 mg/L 6-benzylaminopurine (6-BA), or 14 days with 300 mM NaCl. RNA was separately extracted from the above materials.

Quantitative PCR (qPCR) primers of *LbSAD2* were designed by Beacon Designer Free Edition software (version 7.8) using *LbTUBULIN*-S and *LbTUBULIN*-A as internal references. PCR thermal cycling was as follows: denaturation at 95°C for 5 min, followed by 40 cycles of denaturation at 94°C for 20 s, annealing at 58°C for 15 s, and elongation at 65°C for 15 s. Three replicate biological experiments were performed. The leaves of stages A and B were used as the control of treatments (relative level is 1). The relative expression was calculated according to the formula 2^–△△C(T)^ (Yuan et al., 2019b).

### Transcriptional Activation Assay of *LbSAD2* in Yeast Cells and *in situ* Hybridization of LbSAD2 in *L. bicolor*

The ORF of *LbSAD2* was introduced into the vector pGBKT7/BD using Ndel digestion sites according to the instruction of an In-Fusion HD Cloning Kit (Clontech Laboratories, Inc.). The vectors pGBKT7/BD (empty control), pGBKT7-*LbSAD2* (experimental group), pGBKT7-lam (negative control), and pGBKT7-*LbTTG1* (positive control) were separately transformed into Y2H Gold yeast (*Saccharomyces cerevisiae*) cells using the Yeastmaker Yeast Transformation System 2 (TaKaRa). After an initial 3-day culture on SD/–Trp medium, the transcriptional activity of the yeast was evaluated according to their growth on SD/–Trp medium for 2 days at 30°C (Guo et al., 2013). β-Galactosidase activity was measured based on the growth on SD/–Trp/X-α-gal plates (Han et al., 2019).

In order to verify whether the location of LbSAD2 was related to the salt gland, *in situ* hybridization of LbSAD2 was carried using the developing leaves (the first leaves after germination for 5–8 days) of *L. bicolor* according to Leng et al. (2021). In brief, after fixed with 4% paraformaldehyde and the leaves were embedded in paraffin following gradient alcohol dehydration, 8-μm thin sections were treated with proteinase K, and then hybridized with 6 ng/μl hybridization solution at 37°C overnight. Localization of LbSAD2 digoxin-labeled probe (5′-DIG-GCGAAGACAGAAUCAACACGAACUGGGAGC-3′, purified by HPLC) was detected as blue-violet.

### Construction and Transformation of *p35S:LbSAD2* Into Arabidopsis

The *LbSAD2* ORF was cloned into the vector pCAMBIA3301 under the control of the CaMV 35S promoter to generate *p35S:LbSAD2*, using the primers *LbSAD2* F0 and *LbSAD2* R0 (Supplementary Table 1), according to the instructions of the In-Fusion HD Cloning Kit (Clontech Laboratories, Inc.). The *p35S:LbSAD2* vector was introduced into the *A. tumefaciens* strain GV3101, which was then transformed into Arabidopsis Col-0 and *CS65878* by the *Agrobacterium*-mediated floral dip method (Clough and Bent, 2010). After screening with herbicide for three consecutive generations, homozygous *Col-35S:LbSAD2* and *CS65878-35S:LbSAD2* lines were retained for qPCR.

Plants heterologously expressing *LbSAD2* were identified by PCR using the primers *SAD2*-S and pCAMBIA3301-A after extraction of genomic DNA. Total RNA of several strains of *Col-35S:LbSAD2* and *CS65878-35S:LbSAD2* was then extracted using the FastPure Plant Total RNA Isolation kit (Vazyme, China) according to the manufacturer’s instructions. qPCR was conducted to evaluate the expression level of *LbSAD2* in *Col-35S:LbSAD2* and *CS65878-35S:LbSAD2* using *LbSAD2* RTS and *LbSAD2* RTA primers. Amplification of the *ACTIN2* gene of Arabidopsis was used as an internal control (primers *ACTIN2* sense and *ACTIN2* anti). Three replications were carried out for each transgenic line. Three lines each with high, medium, and low *LbSAD2* expression levels, respectively, of *Col-35S:LbSAD2* and *CS65878-35S:LbSAD2* were used for further experiments.

In the calculation of the expression levels of LbSAD2 in the *Col-35S:LbSAD2* plants, the line OE26 with the lowest expression level was used as the control (relative level is 1) to calculate the relative expression level of different overexpression strains (Leng et al., 2021). The same method was used to calculate the expression level of LbSAD2 in *CS65878-35S:LbSAD2* lines using CL6 as the control.

### Phenotypic Observation of Trichome and Root Hair Development in *Col-35S:LbSAD2* and *CS65878-35S:LbSAD2* Arabidopsis

Phenotypes of transgenic plants were observed in the T_3_ generation, and 1-week-old homozygous seedlings of the T_3_ generation were photographed under a dissecting microscope (Nikon, Japan). The number of trichomes on the first true leaf was counted for 10 plants of each line. The root hairs of 5-day-old *Col-35S:LbSAD2* and *CS65878-35S:LbSAD2* seedlings were also counted. The same root position (0.5 cm from the apex) was selected to count the root hairs with 10 replicates, which was counted using ImageJ software.

### Effect of NaCl Concentration on Germination and Root Length of Transgenic Arabidopsis

Three *Col-35S:LbSAD2* and three *CS65878-35S:LbSAD2* lines with high, medium, and low *LbSAD2* expression levels, respectively, were used for NaCl treatment. All seeds were sown in 1/2 MS medium containing different concentrations of NaCl (0, 50, 100, 150, and 200 mM). After 24 h, the seed germination percentage was calculated based on the number of radicles breaking the seed coats by >1 mm, using the formula: germination percentage (%) = number of germinated seeds/total number of seeds × 100%. The cotyledon growth rate in 3 days was calculated using the formula: cotyledon growth rate (%) = cotyledon seed number/total seed number × 100%. Fifty seeds of each line were sown in each treatment, and three replicates were performed.

All seeds were uniformly sown in a medium with different NaCl concentrations (0, 50, 100, 150, and 200 mM) to observe the effect of NaCl on root growth. Five-day-old seedlings were photographed for root length measurement using ImageJ software. Ten seedlings were measured for each line.

### Measurement of Physiological Indicators in Transgenic Arabidopsis

Four-day-old uniform Arabidopsis seedlings grown in 1/2 MS medium were transplanted into soil for NaCl treatment (0 and 100 mM) after 1-week adaptation. Two-week-old seedlings grown in different NaCl concentrations were pooled to 0.5 g and their contents of Na^+^, K^+^, malondialdehyde (MDA), and proline were measured according to Han et al. (2019) and Guo et al. (2018). Ion concentrations were determined using a flame photometer (M410, Sherwood, United Kingdom). Five replicates were performed for each line.

### RT-qPCR of Genes Related to Trichome Formation and Stress in Transgenic Arabidopsis

Five-day-old Arabidopsis seedlings of all lines grown in 1/2 MS medium were collected for RNA extraction. Quantitative reverse transcription PCR (RT-qPCR) was performed using primers targeting genes related to trichome differentiation, including *GLABRA 1* (*AT3G27920*, *AtGL1*), *WD40 REPEAT-LIKE SUPERFAMILY PROTEIN* (*AT5G24520*, *AtTTG1*), *SUPER SENSITIVE TO ABA AND DROUGHT 2* (*AT2G31660*, *AtSAD2*), *SUPER SENSITIVE TO ABA AND DROUGHT 1* (*AT5G48870*, *AtSAD1*), *GLABRA3* (*AT5G41315*, *AtGL3*), *ENHANCER OF GLABRA3* (*AT1G63650*, *AtEGL*3), *CAPRICE* (*AT2G46410*, *AtCPC*), and *TRYPTICHON* (*AT5G53200*, *AtTRY*), which are listed as genename-S and genename-A (for example, *AtTTG1*-S, and *AtTTG1*-A) in Supplementary Table 1.

RNA extraction was performed on seedlings treated with 0 and 100 mM for 1 week. Three stress-related marker genes were selected for RT-qPCR: *SALT OVERLY SENSITIVE 1* (*AT2G01980*, *AtSOS1*), Δ*1-PYRROLINE-5-CARBOXYLATE SYNTHETASE 1* (*AT2G39800*, *AtP5CS1*), and *GST CLASS TAU 5* (*AT2G2945*, *AtGSTU5*) (Supplementary Table 1). Three biological replicate experiments were performed. Relative expression levels were calculated using the formula 2^–△^^△^^C(T)^. *AtACTIN2* (primers *ACTIN2* sense and *ACTIN2* anti) was used as an internal control.

### Measurement of ABA Sensitivity in Transgenic Arabidopsis

Three *Col-35S:LbSAD2* and three *CS65878-35S:LbSAD2* lines with high, medium, and low *LbSAD2* expression, respectively, were subjected to ABA treatment. All seeds were sown in 1/2 MS medium with different ABA concentrations (0, 0.5, 1.0, 1.5, and 2.0 μM). The seed germination percentage after 24 h and the cotyledon growth rate in 4 days were calculated as described above. Fifty seeds of each line were sown in each treatment, and three replicates were performed. All seeds were uniformly seeded in a medium with different ABA concentrations (0, 0.5, 1.0, 1.5, and 2.0 μM) to observe the effect on root growth. Seven-day-old seedlings were photographed for root length measurement using ImageJ software. Ten seedlings were measured for each line.

### RT-qPCR of Genes Related to ABA Synthesis and Signal Transduction in Transgenic Arabidopsis

RNA extraction was performed on seedlings treated with 0 and 0.5 μM ABA for 1 week. Three ABA marker genes were selected for RT-qPCR: the ABA signal pathway genes *RESPONSIVE TO ABA 18* (*AT1G43890*, *AtRAB18*) and *SNF1-RELATED PROTEIN KINASE 2* (*AT4G33950*, *AtSRK2E*) and the ABA synthesis gene *9-CIS-EPOXYCAROTENOID DIOXYGENASE 3* (*AT3G14440*, *AtNCED3*) (Supplementary Table 1). Three replicate biological experiments were performed. Relative expression levels were calculated using the formula 2^–ΔΔC(T)^. *AtACTIN2* (primers *ACTIN2* sense and *ACTIN2* anti) was used as an internal control.

### Statistical Analysis

Statistical analysis was performed using SPSS with a significance cutoff of *P* = 0.05 (Duncan’s multiple range tests). ANOVA with orthogonal contrasts and mean comparison procedures were used to detect differences between treatments.

## Results

### Cloning and Bioinformatics Analysis of *LbSAD2*

**FIGURE 1:**
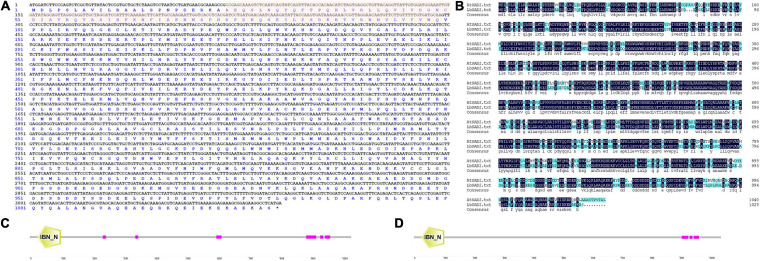
*LbSAD2* encoded importin-β protein. **(A)** Nucleotide and amino acid sequences of *LbSAD2* analyzed with DNAman. The red shadow indicates the importin-β N-terminal (IBN-N) conserved domain. **(B)** DNAman comparison of the *Limonium bicolor* and *Arabidopsis thaliana SAD2* genes. Their percent identity was 73.56%. **(C,D)** The conserved IBN-N domains of LbSAD2 (**C**; located at amino acids 22–99) and AtSAD2 (**D**; located at amino acids 25–102). Pink indicates low-complexity domains. Drawn with SMART.

**FIGURE 2:**
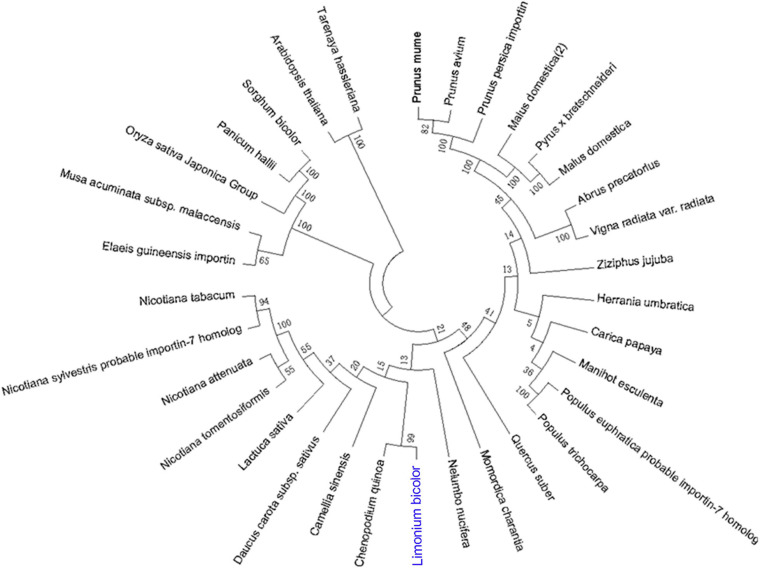
Phylogenetic relationships of plant species based on amino acid sequences of SAD2. The *SAD2* genes of different species were identified using NCBI BLASTp, and then phylogenetic relationships between the corresponding proteins, based on the amino acid sequence of LbSAD2, were reconstructed by the neighbor-joining method using MEGA and ClustalX software.

Based on the full-length sequence from the transcriptome data, we cloned an ORF of 3,090 bp, encoding 1,029 amino acids, for *LbSAD2*. The predicted molecular weight of the protein was 255,012.20 Da, and the isoelectric point (PI) was 4.87. The protein contains 454 hydrophobic and 575 hydrophilic amino acids (Cys + Gly + Thr) (Figure 1A) and is a hydrophilic protein with no transmembrane helix. DNAman analysis detected high sequence similarity between *LbSAD2* and *AtSAD2* (Figure 1B). The amino acid sequence of the LbSAD2 protein included an importin-β N-terminal (IBN-N) conserved domain, located at amino acid positions 24–99 (Figures 1A,C), and the sequence of AtSAD2 also had an IBN-N conserved domain, at amino acids 25–102 (Figure 1D). We also performed an NCBI BLAST analysis using the LbSAD2 protein as the query; a phylogenetic tree of the homologs identified by BLAST showed that LbSAD2 had particularly high similarity to the SAD2 protein of quinoa (*Chenopodium quinoa*), another recretohalophyte (Figure 2).

**FIGURE 3:**
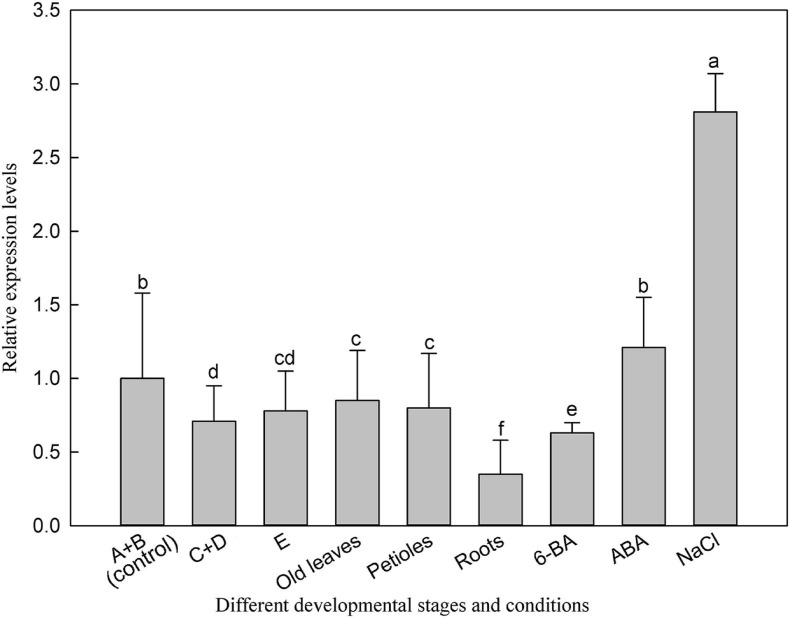
Expression of *LbSAD2* in *L. bicolor* in different developmental stages and conditions. (A) Stage A, undifferentiated, 4–5 days after sowing; (B) stage B, salt gland differentiation, 6–7 days after sowing; (C) stage C, stomatal differentiation, 8–10 days after sowing; (D) stage D, epidermal differentiation, 11–16 days after sowing; (E) stage E, mature, more than 17 days after sowing. Old leaves, leaves >20 days old; petiole, base of leaf in stage E; root, root of seedling in stage E. 6-BA, 1 mg/L 6-BA treatment for 24 h at six-leaf seedlings; ABA, seeds were seeded in MS medium containing 0.1 mg/L ABA for 14 days; NaCl, seeds were seeded in MS medium containing 300 mM NaCl for 14 days. Data are means of three replicates ± SD; different letters indicate significant differences at *P* = 0.05 according to Duncan’s multiple range test.

We then investigated the expression of *LbSAD2* in *L. bicolor* at different developmental stages and under different experimental treatments. *LbSAD2* expression was the highest under NaCl (300 mM) treatment, ABA treatment, and early developmental periods (Figure 3). The lowest expression levels were observed in the root, which implied that *LbSAD2* was induced by NaCl and was highly abundant during the salt gland development stage.

### LbSAD2 Localized in the Cell Membrane, Nucleus, and Salt Gland and No Self-Activation Was Detected

**FIGURE 4:**
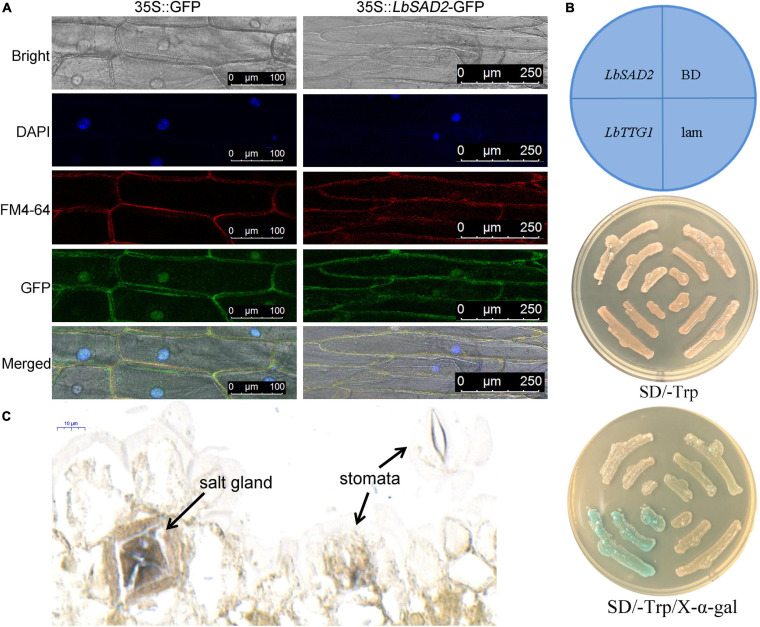
Verification of LbSAD2’s subcellular localization, self-activation, and *in situ* hybridization. **(A)** Subcellular localization analysis in onion epidermal cells expressing *35S:LbSAD2*-GFP. pCAMBIA 1300-35S-sGFP was used as an empty control vector. Bar = 100 μm in 35S:GFP; bar = 250 μm in 35S:*LbSAD2*-GFP. **(B)** Self-activation assay on SD/–Trp medium and SD/–Trp/X-α-gal medium using Y2H Gold yeast. LbSAD2:pGBKT7-LbSAD2, experimental group; LbTTG1:pGBKT7-LbTTG1, positive control; BD, empty vector control (pGBKT7/BD); lam, pGBKT7-lam negative control. **(C)** Digoxin-labeled probe was used for *in situ* hybridization using expanding leaves (germination for 5–8 days).

We determined the subcellular localization of LbSAD2 by detecting the expression of *LbSAD2* fused to the green fluorescent protein (GFP) reporter gene. Using pCAMBIA 1300-35S-sGFP as a control, we found that LbSAD2 was localized to the cell membrane and nucleus (Figure 4A). Next, we incorporated *LbSAD2* into the pGBKT7/BD vector and tested its function by yeast self-activation. The results indicated that *LbSAD2* was not self-activated, indicating that LbSAD2 is not a transcription factor (Figure 4B). By *in situ* hybridization, LbSAD2 was identified in the salt gland and no signal was detected in the stomata and mesophyll cell (Figure 4C), indicating that LbSAD2 may be special to the salt gland.

### *LbSAD2* Heterologously Expressed in Arabidopsis Participates in Trichome and Root Hair Development

**FIGURE 5:**
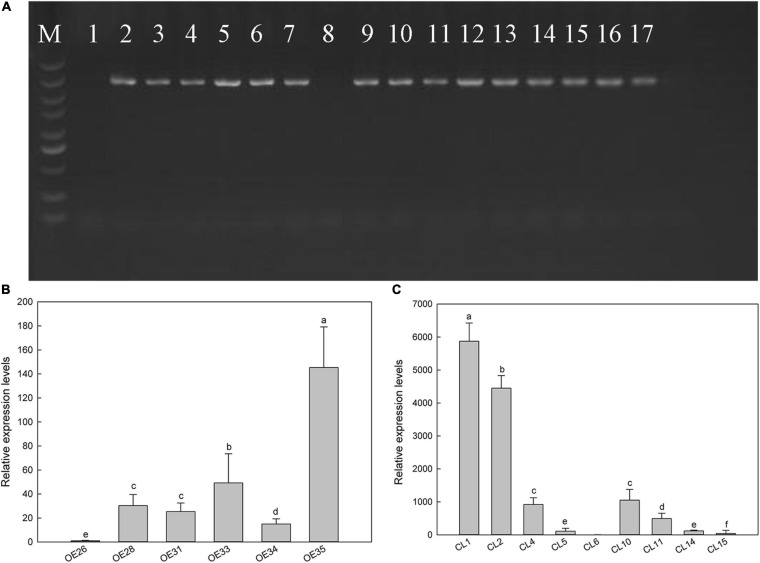
Screening of transgenic Arabidopsis lines by PCR and real-time PCR. **(A)** Electrophoresis of PCR products with Arabidopsis heterologous expression strain DNA as template. M, DNA marker with standard molecular weight of 5,000; lane 1, overexpression negative control (using WT DNA as template); lane 8, mutant negative control (using CS65878 DNA as a template); 2–7, different *Col-35S:LbSAD2* lines; 9–17, different *CS65878-35S:LbSAD2* lines. **(B)** Expression level of *LbSAD2* detected by quantitative PCR in *Col-35S:LbSAD2* lines; OE numbers represent *Col-35S:LbSAD2* overexpression lines. **(C)** Expression level of *LbSAD2* detected by quantitative PCR in *CS65878-35S:LbSAD2* lines; CL numbers represent *CS65878-35S:LbSAD2* complementation lines. Data are means of three replicates ± SD; different letters indicate significant differences at *P* = 0.05 according to Duncan’s multiple range test.

To elucidate the relationship between *LbSAD2* and *AtSAD2* and their roles in epidermal hair formation, we expressed *LbSAD2* heterologously in Col-0 and *CS65878* Arabidopsis. We identified six *Col-35S:LbSAD2* and nine *CS65878-35S:LbSAD2* lines (Figure 5A). The expression level of each plant was identified by RT-qPCR. Six transgenic lines with high, middle, and low expression of *LbSAD2* [*Col-35S:LbSAD2* OE28, OE33, and OE35 (Figure 5B); *CS65878-35S:LbSAD2* CL10, CL2, and CL1 (Figure 5C)] were retained to assess the effect of heterologous expression of *LbSAD2* on trichome and root hair development.

**FIGURE 6:**
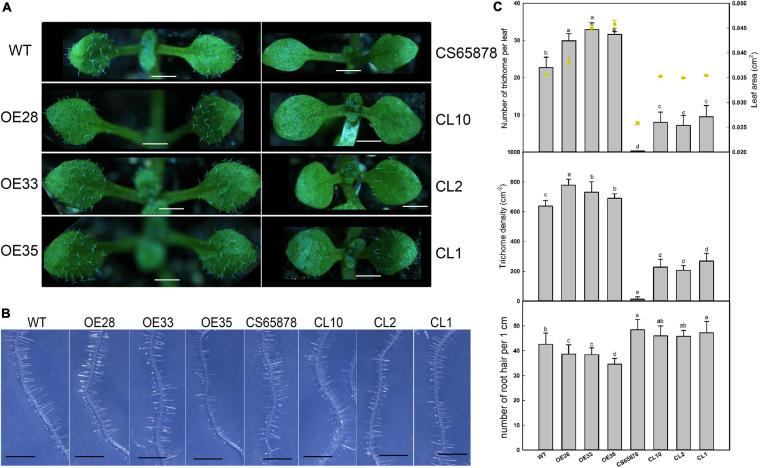
Phenotypic observation of trichome and root hair development in *Col-35S:LbSAD2* and *CS65878-35S:LbSAD2* Arabidopsis lines. **(A)** Trichomes on the first two rosette leaves of WT, CS65878, *Col-35S:LbSAD2* (OE28, OE33, and OE35), and *CS65878-35S:LbSAD2* (CL10, CL2, and CL1). Photographs show seedlings grown in 1/2 MS medium for 5 days. Bar = 0.1 cm. **(B)** Phenotypes of root hairs of all lines grown for 5 days. Photographs show the seedlings growing in 1/2 MS. Bar = 1 mm. **(C)** The total number of trichomes per leaf, leaf area, trichome density, and root hair numbers of WT, CS65878, *Col-35S:LbSAD2*, and *CS65878-35S:LbSAD2*. The trichome number and leaf area are counted with 10 replicates. The trichome density was calculated by trichome number/leaf area. Root hair number calculated by counting hairs in the same region of each root (from 0.5 to 1.5 cm from the root tip) in 10 plants from each line. Data are mean ± SD of 10 plants; different letters indicate significant differences at *P* = 0.05 according to Duncan’s multiple range test.

First, we compared the first true leaf trichomes of the WT, *CS65878*, *Col-35S:LbSAD2*, and *CS65878-35S:LbSAD2* lines. Each *Col-35S:LbSAD2* line showed an increase in the number of epidermal hairs compared with WT plants, and the *LbSAD2* expression level did not have a dose effect on epidermal hair formation, as different *Col-35S:LbSAD2* lines with varying levels of *LbSAD2* expression showed no differences in trichome induction (Figures 6A,C). Among the *CS65878* lines, the first true leaf of the *CS65878* strain showed no trichome formation, but the *CS65878-35S:LbSAD2* strains had restored trichomes, although fewer than the WT. Besides, to avoid the effect of leaf area to the total trichome number, trichome density was calculated and showed the same trends as the total number (Figure 6C).

We also counted root hairs in each line and found that there was a slight increase in *CS65878* mutants than in the WT, and they were least abundant in *Col-35S:LbSAD2* plants. Thus, overexpression of *LbSAD2* in Arabidopsis reduced root hair development to various degrees (Figures 6B,C).

### Expression Levels of Trichome/Root Hair-Differentiation-Related Marker Genes in *LbSAD2* Transgenic Arabidopsis

**FIGURE 7:**
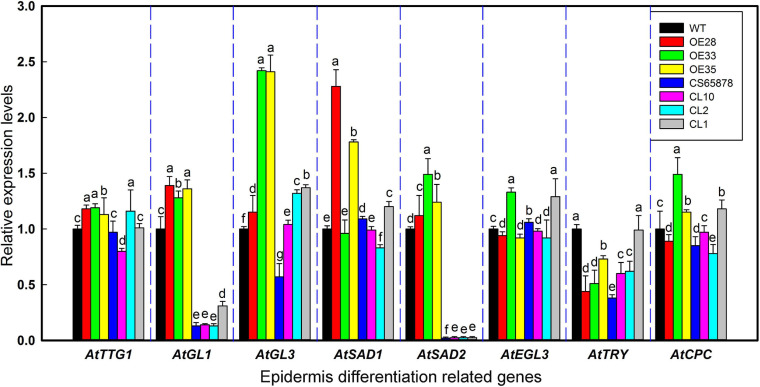
Relative expression levels of *AtTTG1*, *AtGL1*, *AtGL3*, *AtSAD1*, *AtEGL3*, *AtTRY*, *AtCPC*, and *AtSAD2* in transgenic Arabidopsis lines. Five-day-old seedlings were chosen for real-time PCR in different lines. Data are means of three replicates ± SD; different letters indicate significant differences among the same gene at *P* = 0.05 according to Duncan’s multiple range test.

Because the heterologous expression of the *LbSAD2* transgene in WT Arabidopsis increased the abundance of epidermal hairs, we decided to examine the transgene’s effects on the expression of key genes involved in epidermal differentiation, including *AtTTG1*, *AtGL3*, *AtSAD1*, *AtEGL3*, *AtSAD2*, *AtCPC*, and *AtTRY* (Figure 7). We found that the expression of *AtTTG1*, *AtEGL3*, *AtCPC*, and *AtTRY* did not differ significantly between the WT and transgenic lines. However, *AtSAD1*, *AtSAD2*, and especially *AtGL1* and *AtGL3* expression was higher in the *Col-35S:LbSAD2* lines than in the WT. The differences in *AtGL3* expression were the most significant in the comparison between CS65878 and *CS65878-35S:LbSAD2*, indicating that LbSAD2 may work with AtGL3 to initiate the trichome development signal pathway. AtGL3 may be limitedly induced in *CS65878-35S:LbSAD2*, so the increased trichomes were detected after transforming with *LbSAD2* in *CS65878*.

### Effect of NaCl Treatment on *LbSAD2* Transgenic Arabidopsis at Germination and Seedling Stages

**FIGURE 8:**
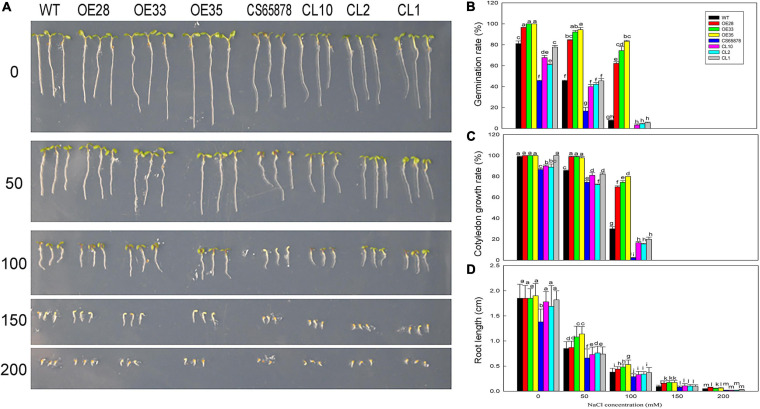
The growth status of *LbSAD2* transgenic Arabidopsis lines during germination at different NaCl concentrations. **(A)** Phenotypes of WT, *Col-35S:LbSAD2* (OE28, OE33, and OE35), *CS65878*, and *CS65878-35S:LbSAD2* (CL10, CL2, and CL1) Arabidopsis grown for 5 days at different NaCl concentrations (0, 50, 100, 150, and 200 mM). **(B)** Germination rates of each line after 24 h at different concentrations of NaCl. **(C)** Cotyledon emergence rates of each line after 3 days at different concentrations of NaCl. **(D)** Root lengths of each line at different concentrations of NaCl. Fifty seeds per line were sown for each treatment, and three biological replicates were performed. The data for percentage germination and cotyledon growth rate are mean ± SD. Root length of 5-day-old seedlings was calculated using ImageJ software. Data for root length are mean ± SD of 10 plants per line; different letters indicate significant differences at *P* = 0.05 according to Duncan’s multiple range test.

Given that root hair number in Arabidopsis was significantly reduced by heterologous expression of *LbSAD2*, we examined whether there exists any effluence on root absorption in ion or ABA. So, in the next section, we measured salt tolerance indicators and ABA sensitivity in the transgenic plants at the germination and seeding stages. Seeds from each transgenic line were sown in media containing different concentrations of NaCl to observe the effects of NaCl on germination (Figure 8A). The germination percentage 24 h after sowing was much higher in the *Col-35S:LbSAD2* lines than in the other strains under treatment with 0, 50, and 100 mM NaCl. Notably, at NaCl concentrations below 100 mM, *CS65878* plants did not germinate successfully, whereas the *CS65878-35S:LbSAD2* lines germinated at a rate similar to that of the WT (Figure 8B).

We also determined the cotyledon emergence rate of each line for 3 days under different NaCl treatments and found that it was higher for the *Col-35S:LbSAD2* lines than for the other lines. At 0 and 50 mM NaCl, all *Col-35S:LbSAD2* seedlings developed cotyledons. At 50 mM NaCl, all strains except *Col-35S:LbSAD2* were affected by salt stress, as evidenced by lower cotyledon growth rate. At 100 mM NaCl, the *Col-35S:LbSAD2* seedlings had significantly higher cotyledon growth than the WT seedlings, and the same trends were observed in *CS65878-35S:LbSAD2* than in *CS65878*, which showed the lowest cotyledon growth rate among the four groups (Figure 8C).

The root lengths of each group showed similar trends under 5 days of different NaCl treatments. At each NaCl concentration, the root length of the *Col-35S:LbSAD2* and *CS65878-35S:LbSAD2* lines was longer than that of the WT and *CS65878* separately, and the root length of *CS65878* was the shortest (Figure 8D).

**FIGURE 9:**
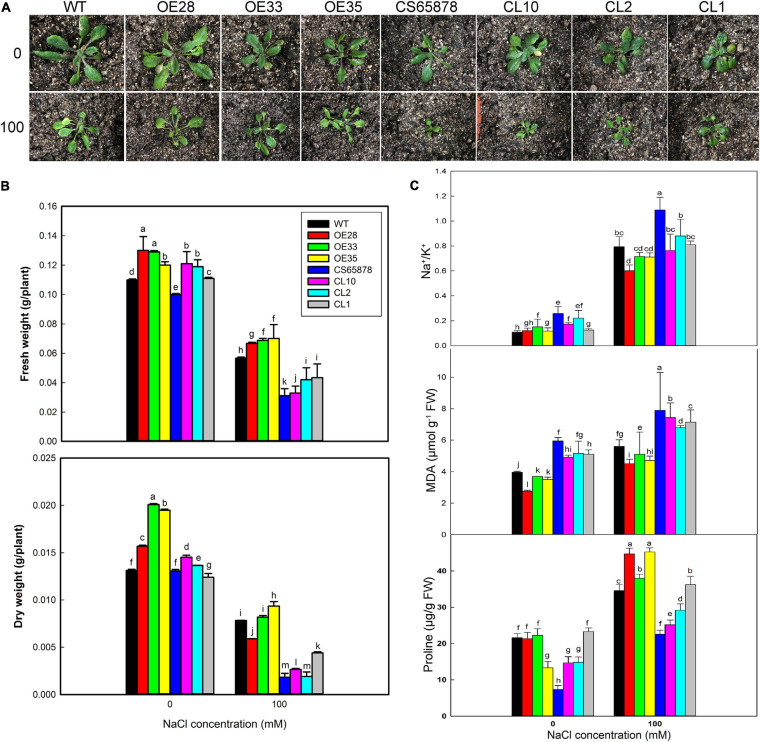
Growth status and determination of physiological indicators of Arabidopsis in 0 and 100 mM NaCl. **(A)** Two-week-old seedlings of WT, *CS65878* (CS65878), *Col-35S:LbSAD2* (OE28, OE33, and OE35), and *CS65878-35S:LbSAD2* (CL10, CL2, and CL1) under 0 and 100 mM NaCl treatment. **(B)** The fresh and dry weight per plant of different lines under 0 and 100 mM NaCl treatments. **(C)** Na^+^/K^+^, contents of MDA, and proline in each line under 0 and 100 mM NaCl treatment. Data are the means of three replicates ± SD; different letters indicate significant differences at *P* = 0.05 according to Duncan’s multiple range test.

Given these consistent trends, we chose the 0- and 100-mM NaCl treatments to use in a further study of the mechanism underlying the increased salt tolerance conveyed by heterologous *LbSAD2* expression at the seedling stage (Figure 9A). We found no significant differences in overall growth trends between the various lines under 0 mM NaCl treatment, but the *CS65878* and *CS65878-35S:LbSAD2* lines grew with most of their rosette leaves laid on the nutrient soil, whereas the WT and *Col-35S:LbSAD2* lines tended to grow upward. In 100 mM NaCl, *Col-35S:LbSAD2* and *CS65878-35S:LbSAD2* plants showed better growth status and biomass than the WT and *CS65878* lines, respectively. The fresh and dry weights quantified the trends (Figure 9B).

Next, we measured several plant physiological indicators (Na^+^, K^+^, proline, and MDA contents) to investigate why the expression of *LbSAD2* can improve salt tolerance. Under 100 mM NaCl treatment, *Col-35S:LbSAD2* lines had higher proline content than the WT. Among the *CS65878-35S:LbSAD2* lines, the CL1 line, which had the highest *LbSAD2* expression, had a proline content approaching that of the WT (Figure 9C). This implies that the heterologous expression of *LbSAD2* can induce the plant to synthesize copious proline, which acts as an organic osmotic adjustment solute, under salt stress. Proline then accumulates in the vacuole, reducing the osmotic potential and allowing the plant to absorb water even at high salt concentration, thus ensuring normal growth and development. The opposite trends were detected in Na^+^/K^+^ and MDA (Figure 9C), which means that the reduced root hair may help absorb less Na^+^ in the transgenic lines than the WT and *CS65878*; thus, less damages led to less MDA production.

### Analysis of Salt-Resistance Marker Gene Expression in *LbSAD2* Transgenic Arabidopsis

**FIGURE 10:**
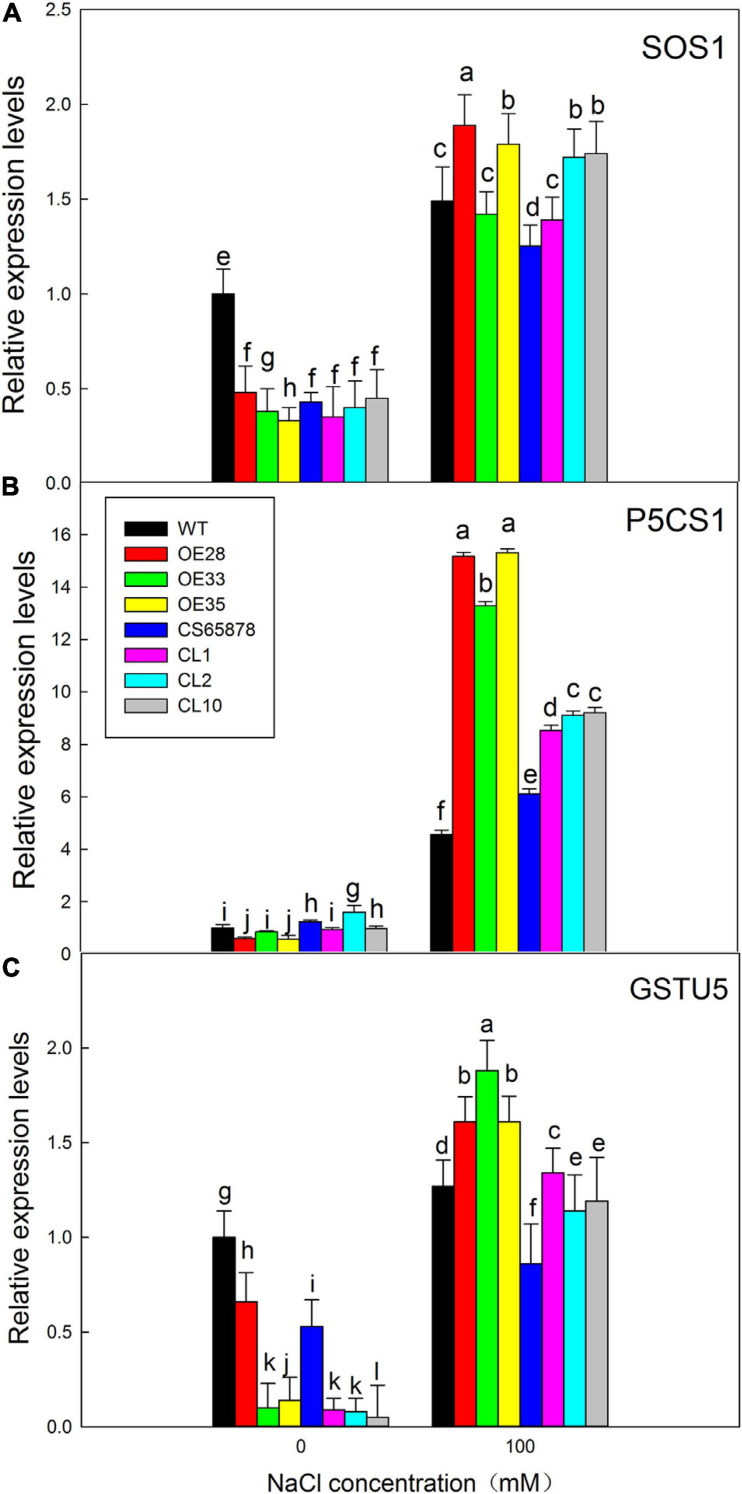
Expression analysis of salt-resistance-related marker genes in *LbSAD2* transgenic Arabidopsis. **(A)** Relative expression levels of *AtSOS1*. **(B)** Relative expression levels of *AtP5CS1*. **(C)** Relative expression levels of *AtGSTU5*. WT, *CS65878*, *Col-35S:LbSAD2* (OE28, OE33, and OE35), and *CS65878-35S:LbSAD2* (CL10, CL2, and CL1) Arabidopsis were grown for 5 days in 1/2 MS with 0 or 100 mM NaCl. Three biological replicates were performed for each gene. Data are means ± SD of three plants; different letters indicate significant differences at *P* = 0.05 according to Duncan’s multiple range test.

We performed RT-qPCR to detect the expression of the three stress-related marker genes, *AtSOS1*, *AtP5CS1*, and *AtGSTU5*, as a way to assess whether differences in their expression might explain the salt tolerance mechanism caused by the transformation with *LbSAD2*. Compared with the control condition, the expression of *AtSOS1* showed no typical trends, which may be due to the less Na^+^ accumulation in transgenic lines, while the other two genes were significantly increased in the salt-treated lines. After NaCl treatment, the expression levels of *AtP5CS1* and *AtGSTU5* in the *Col-35S:LbSAD2* lines were higher than those of WT, with the most significant differences seen for *AtP5CS1* (Figure 10). The same trend was seen in *CS65878-35S:LbSAD2* compared with *CS65878*. Thus, the enhanced salt tolerance produced by transforming Arabidopsis with *LbSAD2* may arise through increases in *AtP5CS1* and/or *AtGSTU5* expression.

### Effect of ABA on the Germination of *LbSAD2* Transgenic Arabidopsis

**FIGURE 11:**
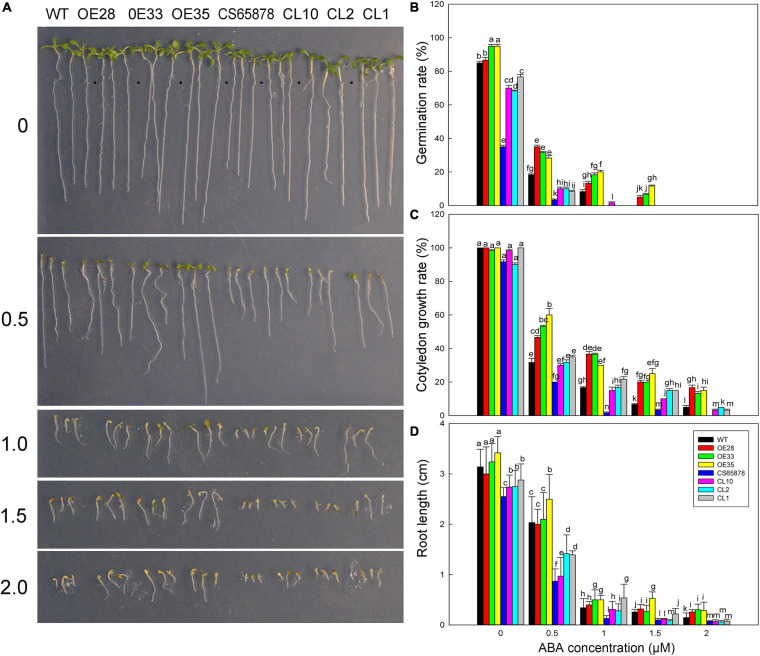
Germination of Arabidopsis transgenic lines at different ABA concentrations. **(A)** Phenotypes of WT, *Col-35S:LbSAD2* (OE28, OE33, and OE35), *CS65878*, and *CS65878-35S:LbSAD2* (CL10, CL2, and CL1) Arabidopsis grown for 5 days at different ABA concentrations (0, 0.5, 1.0, 1.5, and 2.0 μM). **(B)** Germination rates of Arabidopsis lines at different ABA concentrations at 24 h. **(C)** Cotyledon emergence rates of each line at different ABA concentrations at 4 days. **(D)** Root length of each line at different ABA concentrations. Fifty seeds per line were sown for each treatment, and three biological replicates were performed. The data for percentage germination and cotyledon growth rate are mean ± SD. Root length of 7-day-old seedlings was calculated using ImageJ software. Data for root length are mean ± SD of 10 plants per line; different letters indicate significant differences at *P* = 0.05 according to Duncan’s multiple range test.

Given that the *CS65878* mutant is reported to be sensitive to ABA in seed germination and seedling growth (Verslues et al., 2006), we measured the ABA sensitivity of our transgenic lines at the germination stage to observe the effect of ABA on seed germination, cotyledon growth rate, and root length. Seeds of each line were planted in several concentrations of ABA (0, 0.5, 1.0, 1.5, and 2.0 μM), and we assessed their germination percentages after 24 h. In general, the transgenic lines were the least sensitive to ABA, and the *CS65878* mutant was the most sensitive (Figure 11A). No significant difference was detected among the lines at 0 μM, but differences started to appear at 0.5 μM ABA and became more prominent as the ABA concentration increased. The *Col-35S:LbSAD2* lines had a much higher germination rate than the other strains; the *CS65878* lines had the lowest germination rate and did not germinate at all at 2.0 μM ABA, and the *CS65878-35S:LbSAD2* lines had a similar germination rate to the WT (Figure 11B).

At several ABA concentrations, there was basically no cotyledon emergence at 3 days after sowing, so we counted the cotyledon emergence rate of each plant at 4 days. At 1.5 μM ABA, the *CS65878* and *CS65878-35S:LbSAD2* lines essentially did not germinate, but the WT and especially the *Col-35S:LbSAD2* lines showed cotyledon growth. At 2.0 μM ABA, only the *Col-35S:LbSAD2* lines developed cotyledons (Figure 11C). Under the 1.0- and 1.5-μM ABA treatments, few cotyledons were produced, and the 2.0-μM ABA treatment showed no cotyledon growth, but only roots (Figure 11D).

We also measured the root length of each line 7 days after sowing under different ABA treatments. In general, for each treatment, the root length of the *Col-35S:LbSAD2* lines was longer than that of the other lines, the *CS65878-35S:LbSAD2* line was longer than that of the *CS65878* lines, and the root length of the WT was slightly longer than that of the *CS65878-35S:LbSAD2* lines (Figure 11D). Thus, the *Col-35S:LbSAD2* lines clearly grew better and had longer roots than the others at every concentration of ABA.

**FIGURE 12:**
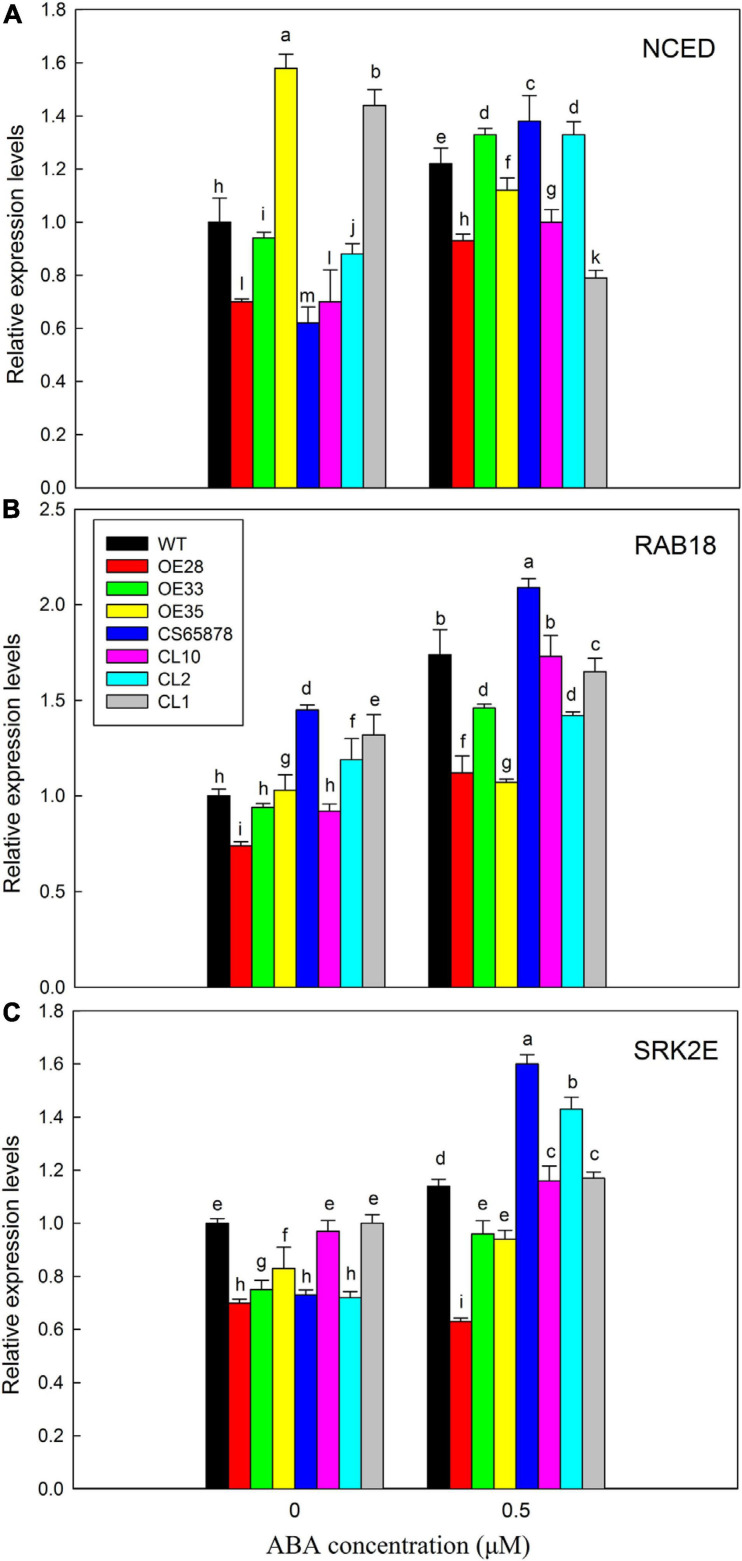
Analysis of expression levels of related marker genes in Arabidopsis in 0 and 0.5 μM ABA. **(A)** Relative expression levels of *AtNCED*. **(B)** Relative expression levels of *AtRAB18*. **(C)** Relative expression levels of *AtSRK2E*. WT, *Col-35S:LbSAD2* (OE28, OE33, and OE35), *CS65878*, and *CS65878-35S:LbSAD2* (CL10, CL2, and CL1) Arabidopsis were grown for 7 days in 1/2 MS with 0 and 0.5 μM ABA. Three biological replicates were performed for each gene. Data are mean ± SD of three plants; different letters indicate significant differences at *P* = 0.05, according to Duncan’s multiple range test.

Based on these results, we chose to use 0 and 0.5 μM ABA for further study of the mechanism by which the expression of *LbSAD2* might reduce the ABA sensitivity of the *CS65878* mutant. We selected three ABA marker genes, *AtRAB18*, *AtSRK2E*, and *AtNCED*, for RT-qPCR testing to test for differences in their expression between the different Arabidopsis lines under ABA treatment. *AtNCED*, which encodes an enzyme important for the synthesis of endogenous ABA, showed no significant expression trends due to ABA treatment in any of the four groups of plants (Figure 12A), indicating that LbSAD2 may not affect endogenous ABA synthesis. However, *AtRAB18* (Figure 12B) and *AtSRK2E* (Figure 12C), two response genes in the ABA signaling pathway that promote seed dormancy when ABA content increases, show expression differences. Under ABA treatment, *AtRAB18* and *AtSRK2E* expression was the lowest in the *Col-35S:LbSAD2* lines and the highest in the *CS65878* lines, which fit with our data showing that the overexpression lines germinated fastest and most reliably under ABA treatment. The *LbSAD2* transgenic lines can profoundly reduce ABA sensitivity by affecting the ABA signal pathway.

## Discussion

Here, we report that the heterologous expression of the gene *LbSAD2* from the recretohalophyte *L. bicolor* enhances salt tolerance in Arabidopsis, through a process involving reductions in root hair development and ABA sensitivity. Though *LbSAD2*, similar to its Arabidopsis homolog *AtSAD2*, encodes an importin-β protein with an IBN-N domain, LbSAD2 showed a significantly different function from AtSAD2 in that AtSAD2 showed no significant role in root hair induction in Arabidopsis, while *LbSAD2* overexpression lines had reduced root hairs and increased trichome, which may be due to the effects of specific domains present only in the *L. bicolor* protein.

Some information was already available about the function of *AtSAD2* in Arabidopsis. Its deletion increases plant sensitivity to ABA, and it encodes a β-domain protein that may participate in nuclear transport and thereby influence plant ABA sensitivity (Verslues et al., 2006). Moreover, an *AtSAD2* mutant shows less DNA damage from UV-B treatment than WT (Zhao et al., 2007). *AtSAD2* is also involved in regulating epidermal hair development; even though the developmental regulatory networks of root hairs and epidermal hairs are closely related (Han et al., 2020b), it had no effect on the number or development of root hairs (Schellmann and Hulskamp, 2005; Gao et al., 2008; Yang and Ye, 2013). However, we found that heterologously expressed *LbSAD2* significantly reduces root hair number in Arabidopsis (Figures 6B,C).

Analysis using a GFP fusion showed that LbSAD2 is localized in the cell membrane and nucleus (Figure 4A), which is consistent with the expression pattern of *AtSAD2* in Arabidopsis (Verslues et al., 2006). This suggested potential avenues for the further study of *LbSAD2* function and expression. In the Arabidopsis epidermal hair development pathway, *AtSAD2* is located relatively upstream, and most upstream genes are generally considered to be transcription factors. However, a yeast self-activation assay to test the function of LbSAD2 showed, unexpectedly, that LbSAD2 (like AtSAD2) was not a transcription factor (Figure 4B). Thus, LbSAD2 may regulate downstream genes by interaction with transcription factors. Besides, as revealed by the transcriptome of different developmental stages (Yuan et al., 2015), the development of the salt gland and trichome may share the same homologous genes. LbSAD2 was located in the salt gland of *L. bicolor* by *in situ* hybridization (Figure 4C), which may be directly related with salt gland development and may explain the function of LbSAD2 in trichome initiation of Arabidopsis.

It is worth mentioning that CS65878 is a double mutant in *AtGL1* and *AtSAD2*, and the mutant behaves with no trichome in the current results which is also consistent with the reported phenotypes (Yoshida et al., 2009; Wasternack and Hause, 2013). Considering that the mutant *sad2* showed no significant difference in trichome number, this indicates that AtSAD2 may not directly regulate trichome development (Yang and Ye, 2013), while GL1 may play a more significant role in trichome differentiation. The current results (Figure 6) of overexpression of *LbSAD2* in CS65878 indeed produced much more trichomes than *CS65878*. Besides, the RT-qPCR results of *AtGL1* (Figure 7) in *Col-35S:LbSAD2* and *CS65878-35S:LbSAD2* indicated that the expression of *AtGL1* was induced after transforming with *LbSAD2* especially in CL10 (with high expression of *LbSAD2*), and this promotes trichome initiation especially in the double mutant CS65878. These may explain why complementation lines had fewer trichomes than WT due to the low expression of *GL1*. What is more, the expression level of *AtSAD2* showed no significant changes between *CS65878* and *CS65878-35S:LbSAD2*, indicating that SAD2 of Arabidopsis had no contribution to the trichome induction of the transgenic lines and GL1 may play more important roles. Moreover, LbSAD2 may coordinate with other pathways (maybe GL3) to regulate trichome initiation. Though the GL1 pathway was blocked to some extent, LbSAD2 can participate in trichome development by other alternative pathways such as combining with GL3 to regulate trichome initiation. The differences between LbSAD2 and AtSAD2 (Figure 1) may be responsive to explain the special function of LbSAD2 in trichome induction.

### Reduced Root Hair Development Promotes Lower Na^+^ Absorption

The fact that the heterologous expression of *LbSAD2* enhances the salt tolerance of Arabidopsis may be explained by two associated changes seen in the overexpression lines: lower Na^+^ accumulation in the roots due to reduced root hair development and lower ABA sensitivity. The heterologous expression of *LbSAD2* in Arabidopsis increased the number of epidermal hairs and decreased the number of root hairs (Figure 6). In the *CS65878* mutant, more root hairs cause more Na^+^ to be absorbed, and the greater Na^+^ accumulation then leads to ion imbalance, resulting in salt sensitivity. Heterologous expression lines have fewer root hairs and less Na^+^ absorption and, therefore, show less MDA production and greater salt tolerance. In addition, we did not observe a dose effect of *LbSAD2* expression on trichome and root hair development, indicating that *LbSAD2* can enhance the function of *AtSAD2* in the positive regulation of trichome formation and negative regulation of root hair development.

According to our RT-qPCR evidence, the heterologous expression of *LbSAD2* directly caused an upregulation of *AtGL3*, thereby regulating the mass production of epidermal hair (Figure 7). Therefore, *AtGL3* was considered to be the downstream of LbSAD2 (in a pathway). In addition, in Arabidopsis, *SAD1* is in the same gene family as *SAD2*, which is also involved in the formation of epidermal hair (Verslues et al., 2006) and which also showed changes in expression level (Figure 7).

Observations of the germination rate and external morphology of plants heterologously expressing *LbSAD2* indicated that the transgene increased their salt tolerance. At 100 mM NaCl, *AtP5CS1* and *AtGSTU5* were more highly expressed in *Col 35S:LbSAD2* than in WT, with the most significant difference seen for *AtP5CS1* (Figure 10). *AtSOS1* encodes a plasma membrane Na^+^/H^+^ antiporter (Qiu et al., 2002), which depends on the transmembrane H^+^ concentration gradient, enters the cell along the electrochemical gradient, and discharges Na^+^ out of the cell, thereby reducing Na^+^ damage (Oh et al., 2009). However, here, no remarkable trends were detected in *AtSOS1*, which may be due to the less Na^+^ accumulation and less exclusion in transgenic lines. Meanwhile, *AtP5CS1* encodes a key rate-limiting enzyme for proline synthesis and, thus, can influence osmotic adjustment by regulating proline synthesis and accumulation (Fabro et al., 2004), and *AtGSTU5* encodes a member of the glutathione S-transferase gene family and is an indicator of antioxidant enzyme system activity (Wagner et al., 2002).

Osmotic adjustment is essential to alleviating the osmotic imbalance caused by salt stress and maintaining cell swelling pressure (Liang et al., 2018). Proline is an important organic osmotic substance (Nounjan et al., 2012), which reduces osmotic potential and prevents plants from losing water. When we measured the proline content of Arabidopsis strains heterologously expressing *LbSAD2*, we found no trend in proline content in the *Col-35S:LbSAD2* lines grown in 0 mM NaCl as compared with the WT. It is noteworthy that in plants grown in 100 mM NaCl, however, the proline content was higher in the *Col-35S:LbSAD2* than in the WT plants, and even higher in the *CS65878-35S:LbSAD2* lines; the CL1 line, which has the highest transgene expression, had a proline content almost as high as the WT (Figure 9). This further demonstrates that the heterologous expression of *LbSAD2* promotes the synthesis of large amounts of proline in the cells, which serves to reduce the osmotic potential, allowing plants to absorb water even at high salt concentration and, thus, ensure their normal growth and development (Nounjan et al., 2012).

### ABA Sensitivity Is Significantly Reduced in *LbSAD2* Transgenic Lines

Abscisic acid is an important plant hormone that regulates many basic biological processes and adaptive responses to various environmental stresses (Umezawa et al., 2010) and plays key roles in seed dormancy and stress resistance. *AtSAD2* encodes a β-domain protein that may be involved in nuclear transport, and *AtSAD2* mutants show increased sensitivity to exogenous ABA (Verslues et al., 2006). We found that the germination of the *CS65878* mutant was sensitive to ABA, consistent with previous reports (Verslues et al., 2006), but the *LbSAD2* transgene conveyed significantly reduced sensitivity to ABA (Figure 11). On the one hand, results of the RT-qPCR experiments indicated that the heterologous expression of *LbSAD2* plays a role in the ABA signaling pathway and in reducing the plants’ sensitivity to exogenous ABA (Figure 12). On the other hand, the reduced root hair number may also be attributed to the reduction of ABA uptake, and insensitivity was detected in the transgenic lines.

In conclusion, Arabidopsis transgenic lines heterologously transformed with *LbSAD2* showed improved salt tolerance during the germination and seedling stages due to reductions in root hair development and ABA sensitivity. The epidermis of the transformed plants showed enhanced trichome development and reduced root hair development, which is different from *AtSAD2* in root hair induction. LbSAD2 may collaborate with AtGL1 and/or AtGL3 to enhance trichome development and reduce root hair differentiation, a possibility that could be further verified using *in vitro* experiments. In addition, the sequence difference between LbSAD2 and AtSAD2 with the former having more low-complexity domains may be directly related to the root hair reduction and salt tolerance of *L. bicolor*. The current evidences showing that LbSAD2 can participate in the trichome development of Arabidopsis and LbSAD2’s specific position in the salt gland of *L. bicolor* strongly support the view of Yuan et al. (2015) that the salt gland and trichome may share a similar evolutionary ancestor. Given that a practicable transformation system exists for *L. bicolor* (Yuan et al., 2014), the role of LbSAD2 in salt gland development can also be further investigated through CRISPR-Cas9 gene editing. Furthermore, elucidating the differences between LbSAD2 and AtSAD2, in regard to upstream genetic and environmental regulation and downstream biochemical/regulatory actions, it is possible to employ this transgene to improve salt tolerance in *Brassicaceae* and ultimately other crop plants.

## Data Availability Statement

The original contributions presented in the study are included in the article/Supplementary Material, further inquiries can be directed to the corresponding authors.

## Author Contributions

FY designed the research. YX and XJ performed the research and wrote the manuscript. YX, XW, and HZ analyzed the data. FY and BW revised the manuscript. All authors contributed to the article and approved the submitted version.

## Conflict of Interest

The authors declare that the research was conducted in the absence of any commercial or financial relationships that could be construed as a potential conflict of interest.

## Footnotes

1 www.megasoftware.net

2 https://web.expasy.org/protparam/

3 https://web.expasy.org/protscale/

4 http://www.cbs.dtu.dk/services/SignalP/

5 https://npsa-prabi.ibcp.fr/cgi-bin/npsa_automat.pl?page=npsa_gor4.html

6 http://smart.embl-heidelberg.de/

7 https://swissmodel.expasy.org/

## Supplementary Material

Supplementary Table 1 Primers used in this work.
