# Supplementary material for: Importin-β From the Recretohalophyte Limonium bicolor Enhances Salt Tolerance in Arabidopsis thaliana by Reducing Root Hair Development and Abscisic Acid Sensitivity
Source: Front Plant Sci. 2021 Jan 13;11:582459. doi: 10.3389/fpls.2020.582459 (PMC7838111; doi:10.3389/fpls.2020.582459)
Supplement: Supplementary Table 1 — Primers used in this work. [file Table_1.DOCX]

Table S1 The primers used in the current paper.

| Primer name | oligonucleotide sequence （5’-3’） |
| --- | --- |
| *LbSAD2*-S | 5-TCCCCTCCTCATCCACTTCTAAACCCT-3 |
| *LbSAD2*-A | 5-AGTAACCACCCATATCACGCCCACAAG-3 |
| *LbSAD2* 1300S | 5-cggggatcctctagagtcgacATGGATCTTCCCAGTCTCGCT-3 |
| *LbSAD2* 1300A | 5-gcccttgctcaccatgtcgacTGAGGCCTTCTCCGCTTTC-3 |
| *LbSAD* RTS | 5-TAGAGCGGAACATAAGGT-3 |
| *LbSAD* RTA | 5-CAGCACGGAGTATAACAG-3 |
| *LbTUBULIN*-S | 5-GGTTGAGTGAGCAGTTCAC-3 |
| *LbTUBULIN*-A | 5-GATAACCAGCCACACCTTAGC-3 |
| *LbSAD2*-F0 | 5-GGACTCTTGACCATGGATCTTCCCAGTCTCGC-3 |
| *LbSAD2*-R0 | 5- CTCAGATCTACCATGGTTGAGGCCTTCTCCGCTTTCT-3 |
| *SAD2*-S | 5-GGAAGTTGCTGAGCTGATTA-3 |
| pCAMBIA3301-A | 5-CGTCGGTTCTGTAACTATCA-3 |
| *Actin*2 sense | 5-GGTAACATTGTGCTCAGTGGTGG-3 |
| *Actin*2 anti | 5-AACGACCTTAATCTTCATGCTGC-3 |
| *AtTTG1*-S | 5-TATTGAGAAGTCTGTTGT-3 |
| *AtTTG1*-A | 5-ATTGTAGAATGTTCCTTATC-3 |
| *AtGL1*-S | 5-CCTTCTTCTTGTCATCAT-3 |
| *AtGL1*-A | 5-ATCATTAGTAGTTGCCATT-3 |
| *AtGL3*-S | 5-GCTTAGATGTGCTTGGAGAG-3 |
| *AtGL3*-A | 5-GAGGATTGAACCGAATGAGAA-3 |
| *AtSAD1*-S | 5-TGGGTGATAATGAAAGGA-3 |
| *AtSAD1*-A | 5-GCCATTGAGTAGAATCTG-3 |
| *AtEGL3*-S | 5-AATCTTCTGGTCTGTCTC-3 |
| *AtEGL3*-A | 5-AATCGTCTTCCTTGTCTT-3 |
| *AtTRY*-S | 5-CTTCTTCTTCTTGTTCGCTCTA-3 |
| *AtTRY*-A | 5-ACGGTCAGTGTTATCCATTAC-3 |
| *AtCPC*-S | 5-TCCGAAGAGGTGAGTAGT-3 |
| *AtCPC*-A | 5-ACGAGTTTATACATCCGAGAA-3 |
| *AtSAD2*-S | 5-GAAGGAGATGAAGCACAA-3 |
| *AtSAD2*-A | 5-ACTCGTCCTCATCACTAA-3 |
| *SAD2*jmS | 5-tcagaggaggacctgcatatgATGGATCTTCCCAGTCTCGCT-3 |
| *SAD2*jmA | 5-ttcggcctccatggccatatgATCATCACCATCCTCATCAGCTT-3 |
| *AtSOS1*-S | 5-TTCATCATCCTCACAATGGCTCTAA-3 |
| *AtSOS1*-A | 5-CCCTCATCAAGCATCTCCCAGTA-3 |
| *AtP5CS1*-S | 5-CAAGATGAGATTACATTCG-3 |
| *AtP5CS1*-A | 5-GGTTATGATGACAGGAAT-3 |
| *AtGSTU5*-S | 5-ATGGCTGAGAAAGAAGAAGTGAAGC-3 |
| *AtGSTU5*-A | 5-TTAAGAAGATCTCACTCTCTCTGCC-3 |
| *AtRAB18*-S | 5-TGGGCAAGAGAGATTTAG-3 |
| *AtRAB18*-A | 5-CTTCGTGTCACATCATATAC-3 |
| *AtSRK2E* -S | 5-CCATGAATGGTTTCTAAAGA-3 |
| *AtSRK2E* -A | 5-GAGGTAATGGTTCAGATTC-3 |
| *AtNCED*-S | 5-CCGACTCATGCTATTCTA-3 |
| *AtNCED*-A | 5-GAGTGATCTGAACTTGGTA-3 |
